# Harms from discharge to primary care: mixed methods analysis of incident reports

**DOI:** 10.3399/bjgp15X687877

**Published:** 2015-11-30

**Authors:** Huw Williams, Adrian Edwards, Peter Hibbert, Philippa Rees, Huw Prosser Evans, Sukhmeet Panesar, Ben Carter, Gareth Parry, Meredith Makeham, Aled Jones, Anthony Avery, Aziz Sheikh, Liam Donaldson, Andrew Carson-Stevens

**Affiliations:** Primary Care Patient Safety Research Group, Cochrane Institute of Primary Care and Public Health; School of Nursing and Midwifery Studies, Cardiff University, Cardiff, UK.; Primary Care Patient Safety Research Group, Cochrane Institute of Primary Care and Public Health; School of Nursing and Midwifery Studies, Cardiff University, Cardiff, UK.; Australian Institute of Health Innovation, University of New South Wales, Sydney, Australia.; Primary Care Patient Safety Research Group, Cochrane Institute of Primary Care and Public Health; School of Nursing and Midwifery Studies, Cardiff University, Cardiff, UK.; Primary Care Patient Safety Research Group, Cochrane Institute of Primary Care and Public Health; School of Nursing and Midwifery Studies, Cardiff University, Cardiff, UK.; Centre for Medical Informatics, Usher Institute of Population Health Sciences and Informatics, University of Edinburgh, Edinburgh, UK.; Primary Care Patient Safety Research Group, Cochrane Institute of Primary Care and Public Health; School of Nursing and Midwifery Studies, Cardiff University, Cardiff, UK.; Institute for Healthcare Improvement, Cambridge, MA, US; assistant professor, Department of Paediatrics, Harvard Medical School, Boston, MA, US.; Australian Institute for Health Innovation, Centre for Health Systems and Safety Research, Macquarie University, North Ryde, Australia.; School of Nursing and Midwifery Studies, Cardiff University, Cardiff, UK.; Faculty of Medicine and Health Sciences, University of Nottingham, Nottingham, UK.; Centre for Medical Informatics, Usher Institute of Population Health Sciences and Informatics, University of Edinburgh, Edinburgh, UK; visiting professor of medicine, Harvard Medical School, Boston, MA, US.; Department of Surgery and Cancer, Imperial College London, London, UK.; Primary Care Patient Safety Research Group, Cochrane Institute of Primary Care and Public Health, Cardiff University, Cardiff, UK; patient safety research lead, Primary and Emergency Care Research Centre Wales; visiting chair of healthcare improvement, Department of Family Practice, University of British Columbia, Vancouver, BC, Canada.

**Keywords:** adverse events, discharge, harm, patient safety, primary care, safety incident reports

## Abstract

**Background:**

Discharge from hospital presents significant risks to patient safety, with up to one in five patients experiencing adverse events within 3 weeks of leaving hospital.

**Aim:**

To describe the frequency and types of patient safety incidents associated with discharge from secondary to primary care, and commonly described contributory factors to identify recommendations for practice.

**Design and setting:**

A mixed methods analysis of 598 patient safety incident reports in England and Wales related to ‘Discharge’ from the National Reporting and Learning System.

**Method:**

Detailed data coding (with 20% double-coding), data summaries generated using descriptive statistical analysis, and thematic analysis of special-case sample of reports. Incident type, contributory factors, type, and level of harm were described, informing recommendations for future practice.

**Results:**

A total of 598 eligible reports were analysed. The four main themes were: errors in discharge communication (*n* = 151; 54% causing harm); errors in referrals to community care (*n* = 136; 73% causing harm); errors in medication (*n* = 97; 87% causing harm); and lack of provision of care adjuncts such as dressings (*n* = 62; 94% causing harm). Common contributory factors were staff factors (not following referral protocols); and organisational factors (lack of clear guidelines or inefficient processes). Improvement opportunities include developing and testing electronic discharge methods with agreed minimum information requirements and unified referrals systems to community care providers; and promoting a safety culture with ‘safe discharge’ checklists, discharge coordinators, and family involvement.

**Conclusion:**

Significant harm was evident due to deficits in the discharge process. Interventions in this area need to be evaluated and learning shared widely.

## INTRODUCTION

One in five patients experiences an adverse event within 3 weeks of discharge from hospital, although the extent and severity of these events are not fully understood.^1^–^4^ With 17.7 million hospital admissions each year in England alone,^5^ there is great potential for significant reduction in harm from even small improvements in this process. The safety of discharge from hospital to the community is a recurring theme in the patient safety literature,^6^–^10^ and a focus for improvement initiatives in the UK,^11^–^16^ and internationally.^17^,^18^ Despite this work, there is a dearth of empirical research to describe the chain of events leading to problems with discharge.

While previous studies have identified *what* is occurring to patients at discharge (such as poor medicines reconciliation and lack of follow-up arrangements), no studies have used patient safety incident reports from primary care to identify *why* they occurred. Understanding the characteristics of incidents and identifying potential contributory and contextual factors, even at a basic level, can inform the development of interventions to improve the quality and safety of clinical practice.^19^–^23^

The National Reporting and Learning System (NRLS) from England and Wales enables learning from a large national database of patient safety incident reports made by front-line healthcare staff. This study aimed to describe patient safety incidents associated with discharge from secondary to primary care reported to the NRLS, to identify contributory factors and associations with harm, and to use these findings to inform recommendations for practice improvement and future research.

**How this fits in**

Up to one in five patients experiences an adverse event within 3 weeks of leaving hospital. Despite 17.7 million hospital admissions a year in England alone, little is known about the harms that occur to patients around discharge and even less about why this occurs. This is the first mixed methods analysis of nationally collected safety incident reports from general practice arising from hospital discharge. Types and severity of harm are described, and contributory factors and priority areas for improvement efforts identified, leading to recommendations for practice.

## METHOD

### Data source

The NRLS is a national reporting system that collates reports about patient safety incidents defined as ‘any unintended or unexpected incident that resulted in or could have resulted in harm to one or more patients receiving state-funded care’.^24^ The NRLS was launched in 2003, receives approximately 100 000 reports a month largely written by healthcare professionals, and it has been mandatory for all organisations in England and Wales to contribute their incident reports since 2010. Reports are received from healthcare organisations in England and Wales, having been generated locally and anonymised. Each report contains categorical information about location, patient demographics, and reporter perception of severity of harm — collected in a structured report form — as well as free-text descriptions of the incident, potential contributory factors, and planned actions to prevent reoccurrence.^22^,^24^ The free-text description, where the reporter is asked to describe what happened and why they think it happened, offers a rich seam of qualitative data for identification of areas for improvement. The perspective given in reports submitted by primary care professionals offer unique insight into the harms occurring in community settings as a result of deficits within the discharge process, which would not be evident in reports submitted from secondary care.

### Study design

Free-text searches for ‘Discharge’ (and related synonyms) and pre-existing filters within the NRLS database were applied to identify a sample from 42 729 general practice reports from England and Wales received by NRLS between April 2003 and June 2012. A retrospective cross-sectional mixed methods study was carried out combining a detailed coding process, frequent generation of data summaries using descriptive statistical analysis, and thematic analysis of a special-case sample of reports. The special-case sample included those which included the four most commonly occurring contributory factor clusters and all reports describing severe harm or death reports. New ideas and hypotheses emerged throughout each step of analysis for later corroboration.

### Data coding

A classification system, aligned with the World Health Organization International Classification for Patient Safety, was developed that incorporates multiple coding frameworks and rules for the application of codes to characterise the sequence of events and contributory factors leading to patient safety incidents. All coders had training in root cause analysis and human factors in health care. Codes were applied to each incident, from two multi-axial coding frameworks to describe the type of safety incident and contributory factors, orientating the coder to the content of the report. These frameworks were empirically developed in-house using an inductive grounded approach,^25^ over a period of several months. Codes were developed based on the types of incidents identified in the reports, following discussion within the coding team, which consisted of physicians and patient safety experts. A detailed coding framework was required to reflect the complexity and nature of incidents in primary care. Existing World Health Organization (WHO) International Classification for Patient Safety definitions of harm severity were used.^26^ Between 1–4 codes were used to describe the incident and to describe the potential contributory factor(s). Nine rules were used to structure the order in which codes were assigned to describe the incident based on the recursive incident analysis method developed by the Australian Patient Safety Foundation.^27^ This allowed modelling of the sequence of events leading to the principal patient safety incident type and potential harm. Thus, where a report described multiple incidents or contributory factors, they were coded in chronological order (using 1–4 codes), to provide a rich description of the report.

A random sample of 20% of the reports were double-coded and Cohen’s κ statistics of the primary incident type calculated to estimate the inter-rater reliability. Discrepancies were resolved by a third reviewer, as well as discussed at a weekly coding team meeting to promote reflexivity within the team. Where the severity of harm stated in the NRLS report conflicted with the incident report free-text description, it was adjusted according to the WHO’s International Classification for Patient Safety definitions.^26^

### Data analysis

To explore the relationship between each incident type and its respective contributory factors, qualitative codes were transformed into dichotomous variables for quantitative analyses, frequency distribution, and cross-tabulation.^19^ All severe harm and death reports, and 151 reports that contained the six commonest clusters of contributory factors, underwent a thematic analysis to provide more in-depth contextual insights into this subset of reports.^28^

The study team discussed quantitative and qualitative analyses, and vignette examples, to raise recommendations for practice. The strengths of recommendations were graded by the US Department of Veterans Affairs classification of strength of recommendations.^29^ Focused literature searches were undertaken to determine whether the concepts and ideas for practice improvement had been described in the literature.

## RESULTS

### Baseline characteristics

In all, 598 out of 644 reports were included in the analysis; 46 incidents were excluded due to reports not describing an actual patient safety incident (*n* = 18), insufficient narrative detail (*n* = 24), or no free-text description available (*n* = 4). Cohen’s κ statistics were calculated for the primary incident type (chronologically closest to the outcome experienced by the patient) at κ = 0.79 (95% confidence intervals = 0.78 to 0.81). Reports were submitted from 116 different locations (for example, health boards in Wales, and formerly primary care trusts in England). Some organisations reported a discharge-related incident once, while others reported over 40 reports, although most organisations reported <10 reports.

Table 1 outlines the definitions of harm used and the number of reports that described each level of harm. Table 2 outlines the main incident types described in the reports with a breakdown of the relationship between harmful and non-harmful events in those categories. The four main opportunities for improvement representing nearly 75% of all reports included: discharge communication issues, principally administration-related (*n* = 151, 25%); reliability and quality of referrals to community care teams (*n* = 136, 23%); provision of medications on discharge (*n* = 97, 16%); and availability of therapeutic adjuncts (care equipment) for safe community care delivery (*n* = 62, 10%).

**Table 1.** Severity of harm described in the reports (*n* = 598)

**Severity of harm**	**Example**	**Reports *n* (%)**
**Unclear:** It is unclear from the free-text description what level of harm has occurred	No discharge letter was sent but no record is made of the effect on the patient	**44 (7)**
**No harm:** Patient outcome is not symptomatic or no symptoms detected and no treatment is required	No discharge letter was sent and no harm occurred to the patient	**91 (15)**
**Low harm:** Patient outcome is symptomatic, symptoms are mild, loss of function or harm is minimal or intermediate but short-term, and no or minimal intervention (for example, extra observation, investigation, review, or minor treatment) is required	No discharge letter was sent and the patient had treatment delayed or needed an appointment to resolve the issue	**381 (64)**
**Moderate harm:** Patient outcome is symptomatic, requiring intervention (for example, additional operative procedure, or additional therapeutic treatment), an increased length of stay, or causing permanent or long-term harm or loss of function	No discharge letter was sent, treatment was delayed and the patient required hospital admission as a result	**78 (13%)**
**Severe harm:** Patient outcome is symptomatic, requiring life-saving intervention or major surgical/medical intervention, shortening life expectancy, or causing major permanent or long-term harm or loss of function	No discharge letter was sent and the patient did not receive appropriate treatment as a result and had a stroke and a permanent reduction in function	**3 (**<**1)**
**Death:** On balance of probabilities, death was caused or brought forward in the short-term by the incident	No discharge letter was sent. GP did not instigate appropriate follow up and the patient died as a result	**1 (**<**1)**

**Table 2.** Principal incident types, their frequency and the proportion of harmful reports

**Incident type: *definition***	**All, *n***	**Harmful, *n* (%)**	**Moderate harm or worse, *n* (%)**
**Administration:** *transfer of patient information (discharge communication)*	151	81 (54)	13 (9)
**Referrals:** *referral of patients from one service to another*	136	99 (73)	20 (15)
**Medication:** *prescribing, dispensing, administration, or monitoring of medications*	97	84 (87)	13 (13)
**Care equipment:** *provision of adjuncts essential to safe care*	62	58 (94)	4 (6)
**Treatment and procedures:** *non-medication treatment decision making or execution*	43	42 (98)	10 (23)
**Diagnosis and assessment:** *process of assessing or diagnosing a patient*	26	24 (92)	16 (62)
**Investigations:** *process of investigating a patient’s condition*	14	13 (93)	3 (21)
**Communication:** *face-to-face or telephone, direct transfer of information relating to patient care*	9	7 (78)	2 (22)
**Other:** *for example issues around transportation*	60	55 (92)	1 (2)

**Total**	598	463 (77)	82 (14)

The most frequent contributory factors were inefficient or poorly followed protocols by staff (*n* = 308, 52%); lack of, or insufficient, organisational protocols (*n* = 184, 31%); and environmental issues such as a lack of equipment availability (*n* = 97, 16%). The degree of harm to the patient was identifiable in 554 (93%) of the 598 included reports, with 44 reports not describing patient outcome at all, and a further 91 describing incidents where the patient was not harmed. Most reports (*n* = 463, 77%) described harm occurring to patients, with 381 (64%) experiencing low harm and 82 (14%) moderate harm or worse (see examples 1 and 2 in Box 1).

Box 1. **Edited extracts of incident reports (salient points highlighted by the authors for illustration)**

**Example 1. Severe harm**

Contacted by Ward **** on **Friday afternoon** to inform us that patient was coming home with a catheter and would need a visit on Monday 19.10.09. Over the weekend patient had a visit from on call GP and district nurse. He was virtually **immobile**, confined to bed, had two grade 2 pressure sores, one on each buttock. He couldn’t eat due to extreme oral thrush. None of those problems were addressed on the **discharge letter**. Patient was also sent home with **no analgesia** despite being on morphine in hospital, and was vomiting virtually all weekend. Has been struggling to tolerate any diet and fluids and developed UTI. Patient had been told to return to hospital on Tuesday 20 October for trial without catheter, but **did not know where to go or what time**. Condition deteriorated and **readmitted** on 21.10.09.

**Example 2. Moderate harm**

**91-year-old** patient was admitted to ********** in June 2009. The patient was then discharged to ******* Nursing Home, who then performed a home assessment and the patient was discharged on 28 September. The district nurse rang on 29 September advising that she felt **discharge was inappropriate**. The room was too small for **equipment (hospital bed, hoist, commode)** and care staff were unable to care for him properly. Apparently the patient was hardly eating or drinking (GP spoke to the patient’s daughter who confirmed this). GP discussed with *** Ward at ******** and intermediate care team who felt that the patient should be **readmitted** to nursing home bed via social services.

**Example 3. Low harm**

The health visitor carried out a primary birth visit following the twins’ discharge from the **Special Care Baby Unit** (SCBU). There was **no discharge letter** with information for the service, or medications required. **No discharge plan**. No **resuscitation training given to the parent**. The mother stated that she was told it would be given before discharge, but that it was not received. Twin discharged on oxygen therapy. **No apnoea monitor**. **No risk assessment** surrounding this twin’s care. **No official referral** to the paediatric community nurse and no involvement pre-discharge. The paediatric community nurse was **not informed of the discharge**. The twins had been cared for over the past 7 weeks in the SCBU. **No liaison had been made with the community staff**.

**Example 4. Low harm**

This patient was discharged from ******** on 24/12/08 having had a **laparotomy and subtotal d2 gastrectomy** for gastric adenocarcinoma on 17/12/08. This man was discharged home **without a referral to district nurses**. His wife is very poorly and expected to cope with his care, medication, and heparin injections. This was a very poor discharge **that could have resulted in readmission** and has been **very stressful** for this couple.

**Example 5. Low harm**

Message received from GP 10.4.10 — patient was discharged from … ward 9/4/10 **late pm** — **no referral sent to child district nurse**. Urinary **catheter** (long-term) in situ. **No advice given** to family re changing bags/care of catheter and **no bags supplied** on discharge. **No information** as to whether district nurse can change catheter.

**Example 6. Low harm**

**A 92-year-old** man was discharged from hospital after being recommenced on warfarin therapy. It **was assumed by** the medical staff on the ward that the GP surgery would take over the monitoring of the patient’s INR. The only correspondence the surgery received was an anticoagulation form with the patient target INR and recent INR (International Normalised Ratio) recordings and warfarin dosages. No **indication for the warfarin was documented or date of discharge**. The surgery only became aware of the patient discharge when a receptionist was contacted to request warfarin. The ward sister was contacted and she felt that as a district nurse had been arranged to take an INR nothing else needed to be done. When questioned about the patient suitability she felt that as the patient was taking a lot of medication in hospital there wasn’t an issue. When asked if the patient suitability to change his dose of warfarin was checked she felt that if there was a problem perhaps his daughter could administer the warfarin.

### Discharge communication

Communication failures were identified in 151 reports, including those that resulted from discharge documents not being sent by hospital teams, were delayed, contained erroneous content, or lacked important clinical information such as diagnosis of a severe, life-threatening illness. Fifty-four per cent (*n* = 81) of these 151 incidents described patient harm, including 9% (*n* = 13) classified as moderate harm or worse. Contributory factors identified were organisational factors (*n* = 84, 14%), for example, discharge letters being lost or delayed; and staff errors (*n* = 58, 10%) from illegible handwriting or missing information in the letter.

Reports captured the complex nature of patients discharged into community care who subsequently experienced significant harm as a result of communication failures. These included patients with a recent complex care history, long inpatient stays in intensive care and special care baby units, or with several comorbidities. Thematic analysis identified community teams, as well as patients and carers, who felt poorly briefed on how to manage the care needs of patients with suprapubic catheters, vacuum-assisted closure dressings, or percutaneous endoscopic gastrostomy. One report described parents who did not receive resuscitation training when their baby was discharged home from the special care baby unit while still receiving oxygen therapy at home (see example 3 in Box 1).

### Referrals to community care teams

Ninety-nine incidents (73%) of 136 reports related to quality and reliability of referrals to community nursing staff, social care, or health visitors resulted in patient harm. Twenty of these reports (15%) related to referrals described moderate harm or worse. Over three-quarters (*n* = 104, 76%) of incidents were due to staff error such as sending an incomplete referral or not recognising a patient would need community care (see example 4 in Box 1). Organisational deficits were responsible in 40% (*n* = 55) of reports resulting from confusing referral criteria, or a difficult to follow referral protocol.

Thematic analysis identified several reports describing practitioners’ confusion in selecting the correct referral method from several available, or insufficient information provided to ensure safe provision of community care. This resulted in patients not receiving medication (such as warfarin or insulin), having dressings unchanged, and surgical wounds or pressure ulcers left untended for days. Failures to reinstate care packages left vulnerable patients without basic care that led to a worsening of their condition and readmission. Patients’ and carers’ aptitude and dexterity for using therapeutic adjuncts were described as inadequate and poorly assessed (see examples 4 and 5 in Box 1).

### Provision of therapeutic adjuncts for safe community care delivery

Fifty-eight (94%) of 62 reports involving non-provision or availability of therapeutic adjuncts resulted in patient harm, with *n* = 4, (7%) of those describing moderate harm or worse. Staff error (*n* = 21, 34%) or work/environmental problems (*n* = 35, 56%) were the contributory issues commonly described. Older patients with complex needs were commonly described being discharged without sufficient supply of therapeutic adjuncts such as urinary catheters, catheter bags, insulin needles, wound dressings, or medication dispensing boxes (see example 5 in Box 1). There was a lack of understanding about the extent to which community nursing teams support the provision of adjuncts.

### Provision of medications on discharge

Eighty-four (87%) of 97 reports described medication provision issues that resulted in patient harm, with *n* = 13 (13%) of those causing moderate harm or worse. Most commonly occurring incidents involved patients who had received the wrong doses of medication, received it at the wrong time, dose-dependent medication not being appropriately monitored in terms of target International Normalised Ratio (INR), or the responsibility for monitoring warfarin not established. This was exacerbated when patients were discharged during weekends and public holidays. The most common contributory factors were staff factors, such as a failure to follow an appropriate protocol (*n* = 74, 76%) or organisational, such as lack of an existing appropriate protocol (*n* = 12, 12%). Time spent by community professionals clarifying with hospital colleagues was complicated by lack of, or incomprehensible, documentation, as well as changing shift patterns of junior medical staff (see example 6 in Box 1).

### Existing interventions identified in the literature

Priority areas and recommendations to improve discharge processes are outlined in Box 2 and were developed following a focused search of the literature and discussions of the main quantitative and qualitative findings within the team.

Box 2. **Possible interventions to improve the discharge process**

**Table:** 

**Intervention**	**Strength as per USDVA classification**	**Current evidence of efficacy**
**Improved quality of discharge communication**		
Electronically generated and transmitted discharge communication outputs	Strong	Yes^33^–^35^
Base new discharge communications on existing pro forma	Strong	Yes^13^,^60^
Patient-controlled records	Intermediate	Some – weak^38^

**Referrals to community teams**		
Single, unified referral process to community nursing services	Strong	No

**Promoting culture of discharge safety**		
Use of patient discharge checklists	Intermediate	Yes^58^,^60^
Discharge coordinators producing individualised discharge plans	Intermediate	Yes^55^,^56^
Include families on ward rounds/discharge planning	Intermediate	Yes^61^,^62^

USDVA = US Department of Veterans Affairs.

## DISCUSSION

### Summary

Deficits are highlighted in the quality of discharge communications, delays or lack of referrals to community care teams, and the availability, provision, and safe use of therapeutic adjuncts and medications at discharge.

Over three-quarters of reports described harm to patients. While most of those harmful incidents were ‘low harm’, they represent issues that still caused considerable inconvenience to patients (for example, additional visits, phone calls, and delayed resolution of their problems). It is evident from the descriptions that without the vigilance and reactivity of primary care staff and sometimes patients and relatives, such ‘low harm’ incidents could have resulted in much greater harm. While 14% of incidents contained descriptions of moderate harm outcomes or worse, these could represent the tip of the iceberg and a larger reservoir of significant harm could be occurring as a result of discharge problems.

This article explores how and why safety incidents occur in the community as a result of deficits in the discharge process. It suggests opportunities to improve the safety of discharge from secondary to primary care. The analysis shows the high importance clinical teams should give the discharge process, since some of the most unwell and vulnerable patients are harmed by shortfalls in this process. This study has highlighted known weaknesses in the interface between primary and secondary care, and a variety of interventions are under way in many healthcare systems. So far interventions have largely been at local levels and require robust evaluation and to be fed into wider improvement initiatives seeking to embed good practice in this area. Perhaps the most important change required is a cultural transformation so that safe discharge planning is given as much priority as evidence-based, effective treatments.

### Strengths and limitations

This is the first mixed methods analysis of safety incident reports from primary care in England and Wales about discharge from secondary to primary care. The reliability of Cohen’s κ indicated that researchers were applying the coding frameworks consistently. It is recognised that incidents are under-reported, can represent only the ‘tip of the iceberg’, and can be limited in narrative content. Only events and factors that were explicitly stated in free-text narratives were coded. The NRLS database, like any incident reporting system, is limited by the quality of the data uploaded on to it, and is particularly affected by under-reporting, selective reporting, and incomplete reporting. The findings should be regarded as essentially inductive, and hypothesis generating, requiring confirmation in further studies.^19^,^28^ The methods are also consistent with other studies which interrogated the NRLS.^30^

The findings reflect a synthesis of the difficulties and challenges faced by patients and primary care professionals around the discharge process, as reported by practising healthcare professionals in England and Wales.

### Comparison with existing literature

NHS England^31^ and Hesselink and colleagues^32^ highlighted that failures in communication processes can account for up to 33% of discharge-related safety incidents. Electronic discharge documentation could mitigate most paper-based administration failures,^33^–^35^ and across the UK countries, a process is under way to support 24-hour e-discharge.^12^,^36^,^37^ Electronic discharge summaries should be based on accepted best practices such as those developed by the Scottish Intercollegiate Guidelines Network,^13^ as well as consensus agreement by primary and secondary care professionals about minimal essential information in discharge summaries. In parallel, patient-held records could aid understanding about a recent hospital stay and follow-up plans.^38^–^40^

Internationally, research examining discharge-related safety incidents has also highlighted the role communication processes play in contributing to these types of incidents. The Threats to Australian Patient Safety study examined anonymous electronic error reports from GPs in New South Wales, and found that communication failures were a feature of 19% of the reports and close to half of these reports related to hospital discharge and other hospital-based communication error.^41^ Specifically, communication errors around medications, especially warfarin, are well described in the literature.^42^–^45^

Poor quality, and sometimes inappropriate, referrals received by district nursing teams are well described,^46^–^50^ and each ‘bad’ referral has been estimated to cause 5 hours of extra work for district nurse teams.^51^ To overcome variability in district nurse referral processes, the development and testing of a single, unified electronic referral process with a baseline minimal information requirement could be considered. Co-production of this system between primary and secondary care professionals could aid with completeness of referral, while promoting understanding of the referral criteria and essential information needs of receiving professionals in the community.

Systematic reviews of staff-led interventions around discharge, while showing reductions in hospital stays and readmission rates, have not found strong evidence for effectiveness in improving mortality, health outcomes, and cost.^52^,^53^ However, promoting a culture of safety around discharge through increasing visibility of those responsible for overseeing the process, such as discharge coordinators, has been shown to improve the quality and safety of the discharge process.^54^–^56^ Similarly, methods to promote family involvement in ward rounds have been described, as well as efforts to encourage patient involvement via leaflets and videos given to promote understanding of tests, diagnoses, and follow-up requirements.^57^–^62^ A safety checklist process could be developed to incorporate the prompt initiation of many of those options, including important pathways such as medical reconciliation and INR monitoring,^59^,^63^,^64^ or initiating conversations with patients and family before discharge about the availability and safe use of medicines and therapeutic adjuncts at home.

### Implications for practice

Based on the assessment of existing interventions to improve discharge in practice, the authors would recommend the development and testing of unified, electronic referral and discharge systems co-developed between front-line staff from primary and secondary care, and the promotion of a culture of safety around discharge planning. A safer discharge checklist could initiate conversations with patients and their families around their needs at home. Important common pathways, such as INR monitoring and district nurse visits, should be identified for safe transitioning to the community for patients with comorbidities, and special-case needs should be queried, for example, the preparation of family members to safely deliver medicine or feeds via percutaneous endoscopic gastrostomy. These recommendations are summarised in Box 2.

Quality improvement methods are needed to adapt and localise interventions that have been found to improve the discharge process. Front-line professionals need to be supported by teams in organisations with evaluation expertise to determine how those interventions work best and in which contexts.
